# Genetic Variation in the Platelet Endothelial Aggregation Receptor 1 Gene Results in Endothelial Dysfunction

**DOI:** 10.1371/journal.pone.0138795

**Published:** 2015-09-25

**Authors:** Adam S. Fisch, Laura M. Yerges-Armstrong, Joshua D. Backman, Hong Wang, Patrick Donnelly, Kathleen A. Ryan, Ankita Parihar, Mary A. Pavlovich, Braxton D. Mitchell, Jeffrey R. O’Connell, William Herzog, Christopher R. Harman, Jonathan D. Wren, Joshua P. Lewis

**Affiliations:** 1 Division of Endocrinology, Diabetes, and Nutrition, and Program for Personalized and Genomic Medicine, University of Maryland School of Medicine, Baltimore, Maryland, United States of America; 2 Division of Cardiology, Department of Medicine, Johns Hopkins University School of Medicine, Baltimore, Maryland, United States of America; 3 Department of Obstetrics, Gynecology, and Reproductive Sciences, University of Maryland School of Medicine, Baltimore, Maryland, United States of America; 4 Department of Biochemistry and Molecular Biology, University of Oklahoma Health Science Center, Oklahoma City, Oklahoma, United States of America; 5 Program in Arthritis & Clinical Immunology Research, Oklahoma Medical Research Foundation, Oklahoma City, Oklahoma, United States of America; Case Western Reserve University, UNITED STATES

## Abstract

Platelet Endothelial Aggregation Receptor 1 (PEAR1) is a newly identified membrane protein reported to be involved in multiple vascular and thrombotic processes. While most studies to date have focused on the effects of this receptor in platelets, PEAR1 is located in multiple tissues including the endothelium, where it is most highly expressed. Our first objective was to evaluate the role of PEAR1 in endothelial function by examining flow-mediated dilation of the brachial artery in 641 participants from the Heredity and Phenotype Intervention Heart Study. Our second objective was to further define the impact of PEAR1 on cardiovascular disease computationally through meta-analysis of 75,000 microarrays, yielding insights regarding PEAR1 function, and predictions of phenotypes and diseases affected by PEAR1 dysregulation. Based on the results of this meta-analysis we examined whether genetic variation in *PEAR1* influences endothelial function using an *ex vivo* assay of endothelial cell migration. We observed a significant association between rs12041331 and flow-mediated dilation in participants of the Heredity and Phenotype Intervention Heart Study (P = 0.02). Meta-analysis results revealed that *PEAR1* expression is highly correlated with several genes (e.g. *ANG2*, *ACVRL1*, *ENG)* and phenotypes (e.g. endothelial cell migration, angiogenesis) that are integral to endothelial function. Functional validation of these results revealed that *PEAR1* rs12041331 is significantly associated with endothelial migration (P = 0.04). Our results suggest for the first time that genetic variation of *PEAR1* is a significant determinant of endothelial function through pathways implicated in cardiovascular disease.

## Introduction

Platelet endothelial aggregation receptor 1 (PEAR1; also known as JEDI and MEGF12) is a recently identified transmembrane receptor expressed in a number of different tissues, with highest expression in endothelial cells and megakaryocytes [1]. While little is currently known regarding the molecular mechanism(s) of this receptor, prior investigations suggest that PEAR1 is important in a diverse range of biological functions, including sustained platelet aggregation through glycoprotein αIIbβ3 [2], altered megakaryopoiesis and thrombopoiesis via PI3K/PTEN pathways [3], and apoptotic neuron clearance through endocytosis-dependent activities in dorsal root ganglia [4]. In addition to these mechanism-based investigations, several studies have examined the role of genetic variation in *PEAR1*, most notably the intronic single nucleotide polymorphism (SNP) rs12041331. These studies have implicated rs12041331 genotype in differential *PEAR1* expression as well as platelet aggregation, both at baseline and in the presence of therapeutic agents such as aspirin and prasugrel [5–11]. However, a seemingly paradoxical effect of rs12041331 on cardiovascular phenotypes has been observed; the allele associated with better aspirin response, as measured by platelet function testing, is also associated with higher adverse cardiovascular event rates in patients with coronary artery disease on aspirin, potentially suggesting an alternative role for PEAR1 in cardiovascular disease progression [10].

Given that *PEAR1* is most highly expressed in endothelial cells [1], we first explored the effects of genetic variation in *PEAR1* on endothelial function. Specifically, we tested the impact of rs12041331 on flow-mediated dilation (FMD) of the brachial artery in 641 participants of the Heredity and Phenotype Intervention (HAPI) Heart Study. In an attempt to further define the role of PEAR1 in cardiovascular biology we used a bioinformatics approach called GAMMA (Global Microarray Meta-Analysis) [12] to identify genes consistently correlated with *PEAR1* expression across 75,000 human 1-color microarray experiments from within the publicly available datasets in National Center for Biotechnology Information’s Gene Expression Omnibus. Based on our meta-analysis’s results, we extended our findings by evaluating and confirming the effect of the *PEAR1* rs12041331 variant on endothelial cell migration using functional *ex vivo* assays of human umbilical vein endothelial cells (HUVECs) derived from de-identified umbilical cords.

## Materials and Methods

### HAPI Heart Study Participants

The HAPI Heart Study recruited 868 healthy Old Order Amish (OOA) participants aged 20 years or older from 2003 to 2006 as previously described [13]. This report evaluates 641 HAPI Heart Study participants in whom brachial artery FMD measurements were recorded. Briefly, all study participants discontinued the use of medications, vitamins, and supplements 7 days prior to their initial clinic visit. Physical examinations, anthropometric measures, medical and family histories, and other phenotype information were collected at the Amish Research Clinic in Lancaster, Pennsylvania after an overnight fast. Individuals were excluded if any of the following criteria were met: pregnancy, coexisting malignancy, severe hypertension (blood pressure > 180/105 mmHg), serum creatinine > 2.0 mg/dl, AST or ALT greater than twice the upper limit of normal, hematocrit < 32%, TSH < 0.4 or > 5.5 mIU/l, or inability to safely discontinue prescription and nonprescription medications.

Complete blood count and serum lipid concentrations were assayed by Quest Diagnostics (Horsham, Pennsylvania), and levels of LDL-cholesterol were calculated using the Friedewald equation. Any participant with an LDL-cholesterol greater than 160 mg/dl or taking prescription cholesterol-lowering medications was designated hyperlipidemic. Individuals were described as hypertensive if they had one or more of the following criteria: systolic blood pressure (SBP) ≥ 140 mmHg, diastolic blood pressure (DBP) ≥ 90 mmHg, or requirement of prescription blood pressure lowering medications. Diabetes and current smoking status (cigarette, cigar, or pipe) were obtained by self-report.

Study protocols were approved by the Institutional Review Board at the University of Maryland School of Medicine, and the study was conducted according to the principles expressed in the Declaration of Helsinki. Written informed consent was obtained from each HAPI Heart Study participant; participants were compensated for their participation. Data from the HAPI Heart Study pertaining to the measurements used for analysis are available upon request to preserve the anonymity of OOA participants.

### Flow-Mediated Dilation (FMD)

Assessment of endothelial function was evaluated by FMD of the brachial artery. All brachial artery measurements were obtained after an overnight fast. Briefly, the subject’s left arm was immobilized in the extended position and the left brachial artery was imaged above the antecubital fossa in the longitudinal plane by continuous 2D gray-scale imaging with an 11 MHz ultrasound (HDI 5000CV [Phillips, Andover, Massachusetts]) by a trained sonographer. A baseline rest image was acquired and blood flow was estimated by time-averaging the pulsed Doppler velocity signal obtained from a mid-artery sample volume. Arterial occlusion was created by cuff inflation to suprasystolic pressure (50 mmHg above systolic pressure) for 5 minutes, after which the cuff was deflated. The longitudinal image of the artery was recorded continuously from 30 seconds before to 2 minutes after cuff deflation. Flow images were captured on videotape, and read in a blinded fashion. From longitudinal images, the boundaries for diameter measurement were identified manually with electronic calipers at the lumen-intima interface. Five evenly spaced arterial diameter measurements were taken within a 5 cm segment of vessel at baseline and one minute after cuff deflation, and averaged for the brachial artery width measurement.

### Genotyping

*PEAR1* rs12041331 SNP genotyping in HAPI Heart Study participants was performed using a TaqMan SNP genotyping assay (Applied Biosystems/Life Technologies, Foster City, California). The mean genotype concordance rate for this polymorphism in a subset of duplicate samples was 100% and the genotype call rate was 98.3%.

### GAMMA (Global Microarray Meta-Analysis)

GAMMA [12] was used to identify consistent transcriptional correlations across 75,000 publicly available one-color microarrays from the National Center for Biotechnology Information’s Gene Expression Omnibus (GEO) database in order to identify a set of 30 genes highly correlated with *PEAR1* across a broad range of experimental conditions. Microarrays were quantile-normalized and processed with an automated quality checking process that includes comparison of parametric expression distributions of individual experiments to expected distributions. Unlike traditional meta-analytic approaches which evaluate gene expression under specific experimental conditions, and control for cell or tissue type, GAMMA utilizes heterogeneous conditions in order to identify general co-expression patterns to more accurately identify common biological responses. In other words, its goal is to identify the strongest gene-gene correlations regardless of experimental condition, tissue, or other variables. Therefore, examination of *PEAR1* co-expression patterns in specific cell types (e.g. endothelial cells) or conditions was not explored. The set of genes highly correlated with *PEAR1* can then be considered as related to it in a broad, biological sense and queried for statistically significant biological associations they may share. This approach is useful for genes with little or no annotation. Because the use of Gene Ontology for this purpose has fallen under recent criticism [14] and Gene Ontology covers only biological processes, molecular functions, and cellular components, we also used literature mining [15] to identify published commonalities for the genes that were most highly correlated with *PEAR1* and had entries in MEDLINE (25 of 30 genes); this includes other categories such as disease relevance, phenotype, and other genes predicted to be relevant to *PEAR1*’s genetic neighborhood. GAMMA’s performance has been previously benchmarked by predicting Gene Ontology annotations for 5,000 genes and then comparing the predictions to their known Gene Ontology annotations [12, 16]. Importantly, predicted phenotypes and functions from GAMMA have also been validated experimentally in several published studies [16–21].

### Cell Culture

Fifty-five de-identified umbilical cords were obtained from the University of Maryland Medical Center Division of Maternal and Fetal Medicine under an IRB exempt protocol due to their de-identified nature. Upon receipt of umbilical cords, HUVECs were harvested as described previously [22]. HUVECS were maintained at 37°C in a 5% CO_2_ incubator using Endothelial Basal Media 2 (Lonza, Catalog #CC-3156 & CC-4176, Walkersville, Maryland) containing 2% FBS, 0.04% hydrocortisone, 0.4% hFGF, 0.1% VEGF, 0.1% R3-IGF, 0.1% ascorbic acid, 0.1% hEGF, 0.1% gentamicin-amphotericin-B [GA-1000], and 0.1% heparin. DNA from each cell line was extracted using a Gentra Puregene Cell Kit (Qiagen, Valencia, California) as recommended by the manufacturer. *PEAR1* rs12041331 genotype was determined using a TaqMan SNP genotyping assay (Applied Biosystems/Life Technologies, Foster City, California), which resulted in the identification of 28 major allele homozygotes (GG), 25 heterozygotes (GA), and 2 minor allele homozygotes.

### Endothelial Cell Migration Assay

We evaluated the impact of *PEAR1* rs12041331 genotype on *ex vivo* endothelial cell migration using methods described previously [23]. Briefly, 10 primary HUVEC lines were randomly chosen within each genotype (4 rs12041331 major allele homozygotes [GG], 4 heterozygotes [GA], and 2 minor allele homozygotes [AA]) and split at passage 3 onto a gelatin-coated 6-well plate containing Endothelial Basal Media 2. Confluent HUVEC monolayers were scraped in a uniform manner using a P1000 pipette tip to generate the scratch, which was followed by replacement of growth media. Cells were photographed at 0 and 6 hours using an Axiocam MRc 5 camera (Carl Zeiss Microscopy, Pleasanton, California) mounted on a Lumar.V12 microscope (Carl Zeiss Microscopy, Pleasanton, California). The area of the scratch was measured using ImageJ [24] and used to calculate endothelial cell migration as described in the Statistical Analysis section.

### Statistical Analyses

#### HAPI Heart Study

Summary statistics and frequencies for the OOA HAPI Heart Study were calculated using SAS version 9.2 (SAS Institute Inc., Cary, North Carolina). Measures of Hardy-Weinberg equilibrium were calculated using a χ^2^ test. For HAPI Heart Study-related analyses, P-values less than 0.05 were considered statistically significant. All statistical tests were 2-sided.

Clinical correlates of FMD response were evaluated using a regression-based approach as implemented in SOLAR version 4.07 (Texas Biomedical Research Institute, San Antonio, Texas). Given the unique ancestral history of the Lancaster OOA community, all participants are related and their relationships were accounted for using the extensive genealogical records of the OOA [25] by including a polygenic component as a random effect as previously described [26]. Triglyceride levels were logarithm-transformed for analysis and back-transformed for presentation. The relationship between smoking and FMD was only measured in sex-stratified analyses to account for the OOA community’s cultural norms that limit smoking to males. Association analyses with *PEAR1* rs12041331 and FMD were performed under an additive model using a variance component method that assesses the effect of genotype on the quantitative trait. Analyses were adjusted for age, sex, body mass index (BMI), diabetes, SBP, DBP, and the aforementioned polygenic component, which was modeled using the relationship matrix derived from the complete OOA pedigree structure available through the Anabaptist Genealogy Database [25, 27]. Secondary analyses were adjusted for the same covariates above in addition to baseline brachial artery width (D_base_) and heart rate (HR) to account for changes in FMD caused by these variables [28]. Heritability of FMD response corresponds to the proportion of the trait variance accounted for by the polygenic component, and the heritability estimate was created with adjustments for age and sex.

#### GAMMA

Statistical analyses implemented by GAMMA have been previously described [12, 29]. Briefly, 1-color microarrays were processed to create a gene vs experiment expression matrix in which subsets can be extracted to perform meta-analyses and identify gene-gene correlations. Function, phenotype and disease relevance are predicted by identifying a set of 30 genes that are most correlated in their expression patterns with a query gene of interest. Of these 30 co-expressed genes, 25 had MEDLINE entries and were analyzed by literature-mining software [15] for what they have in common (essentially a “guilt by association” approach). Microarrays were quantile normalized and noise thresholds were used to identify transcription levels that were statistically significant.

#### Endothelial Cell Migration

Each of 10 HUVEC lines was plated into 6-well plates, and 3 equidistant photographs were taken per well at 0 and 6 hours after scratch generation of the endothelial monolayer, resulting in 18 area measurements per cell line at each time point. Mean endothelial cell migration distance was calculated by dividing the area of the scratch by the height of the frame. Differences in endothelial cell migration distance between *PEAR1* rs12041331 genotype groups were assessed using two-tailed analysis of variance. Genotype-specific differences in endothelial cell migration distance were assessed 0 and 6 hours post-scratch generation. P-values < 0.05 were considered statistically significant.

## Results

We evaluated the effect of *PEAR1* rs12041331 on *in vivo* endothelial function in 641 subjects of the HAPI Heart Study. Characteristics of the HAPI Heart Study participants are shown in Table 1. Subjects were generally healthy, middle-aged (mean age = 43.2 years), drug-naïve, and had low prevalence of disease (e.g. diabetes [0.78%], hypertension [12.8%], hypercholesterolemia [16.9%]), and obesity [mean BMI = 26.3]). FMD was normally distributed in this population (S1 Fig). Poorer FMD response was associated with increasing age (0.9% of the variance; P = 0.007), male sex (17.6% of the variance; P = 3.53 x 10^−30^), increased D_base_ (29.2% of the variance; P = 2.5 x 10^−54^), increased DBP (0.6% of the variance; P = 0.026), increased SBP (1.5% of the variance; P = 0.001), decreased HR (7.5% of the variance, P = 2.73 x 10^−12^), and presence of hypertension (0.7% of the variance; P = 0.025) (Table 2). Initially, smoking was also significantly associated with FMD (1.9% of the variance; P = 1.38 x 10^−3^), but after stratifying for sex, since only men smoke in the Amish community, no association was observed (P = 0.817). Also, we found that the variance caused by sex was driven by D_base_, as the effect of sex was diminished (P = 0.85) when adjusting for D_base_. The estimated residual heritability of FMD after adjustment for age and sex was 0.16 ± 0.09 (P = 0.03).

**Table 1:** Characteristics of HAPI Heart Study Participants.

Characteristic (Units)	Men	Women
Number (n)	365	276
Age ± SD (years)	42.2 ± 13.7	44.5 ± 14.1
BMI ± SD (kg/m^2^)	25.6 ± 3.2	27.3 ± 4.8
Systolic blood pressure ± SD (mm Hg)	122.0 ± 12.8	120.4 ± 16.3
Diastolic blood pressure ± SD (mm Hg)	78.0 ± 8.7	75.4 ± 8.4
No. with hypertension (%)^*^	42 (11.5)	40 (14.5)
Total cholesterol ± SD (mg/dl)	202.3 ± 44.7	212.7 ± 48.4
LDL cholesterol ± SD (mg/dl)	136.4 ± 40.9	139.6 ± 45.8
HDL cholesterol ± SD (mg/dl)	53.3 ± 13.0	59.0 ± 14.4
Triglycerides ± SD (mg/dl)^†^	62.8 ± 37.9	70.9 ± 45.5
No. with hypercholesterolemia (%)^‡^	57 (15.7)	51 (18.6)
No. with self-reported diabetes (%)	3 (0.8)	2 (0.7)
Hematocrit ± SD (%)	43.2 ± 2.5	38.5 ± 2.5
White blood cell count ± SD (n x 1000)	5.4 ±1.2	5.2 ± 1.0
Platelet count ± SD (n x 100,000)	231.4 ± 52.3	240.8 ± 50.5
No. of current smokers (%)^§^	73 (20.2)	0 (0)
No. taking aspirin (%)	14 (3.8)	5 (1.8)
No. taking lipid-lowering medications (%)	5 (1.4)	2 (0.7)
No. taking anti-hypertensive medications (%)	1 (0.3)	0 (0)
Brachial artery width pre-occlusion ± SD (mm)	4.1 ± 0.5	3.1 ± 0.4
Brachial artery width post-occlusion ± SD (mm)	4.4 ± 0.5	3.5 ± 0.4

Abbreviations: BMI, body mass index; HAPI, Heredity and Phenotype Intervention; HDL, high-density lipoprotein; LDL, low-density lipoprotein; SD, standard deviation.

SI conversion factors: To convert HDL-cholesterol, LDL-cholesterol, and total cholesterol values to mmol/L, multiply by 0.0259; triglycerides to mmol/L, multiply by 0.0113.

^*^Defined as systolic blood pressure greater than 140 mm Hg or diastolic blood pressure greater than 90 mm Hg or taking prescription medication for previously diagnosed hypertension.

^†^Logarithm-transformed for analysis and back-transformed for presentation.

^‡^Defined as LDL-cholesterol greater than 160 mg/dl or taking prescription medication for previously diagnosed hypercholesterolemia.

^§^Self-reported history of smoking cigarette, pipe, or cigar. Only men report smoking in the OOA community.

**Table 2:** Predictors of Variance in Flow-Mediated Dilation in HAPI Study Participants.

Predictor (Units)	Beta	Standard Error	P-value	Variance of Significant Predictor (%)
Age (years)	-0.05	0.02	0.007	0.9%
Sex (female)	5.07	0.42	3.5 x 10^−30^	17.6%
BMI (kg/m^2^)	0.04	0.06	0.448	
Current smoking (%)^*^	0.15	0.66	0.819	
Self-reported diabetes (%)	-4.72	2.66	0.076	
Brachial artery width pre-occlusion (mm)	-0.65	0.04	2.5 x 10^−54^	29.2%
HDL cholesterol (mg/dl)	0.02	0.02	0.369	
LDL cholesterol (mg/dl)	0	0.01	0.937	
Total cholesterol (mg/dl)	0	0.01	0.578	
Triglycerides (mg/dl)				
Diastolic blood pressure (mm Hg)	-0.06	0.03	0.026	0.6%
Systolic blood pressure (mm Hg)	-0.05	0.02	0.001	1.5%
Mean arterial pressure (mm Hg)	-0.07	0.02	0.003	1.2%
Heart rate (beats/min)	0.17	0.02	2.7 x 10^−12^	7.5%
Hypertension (%)	-1.54	0.68	0.025	0.7%
rs12041331 genotype (A allele)	1.22	0.62	0.047	0.5%

Abbreviations: BMI, body mass index; HAPI, Heredity and Phenotype Intervention; HDL, high-density lipoprotein; LDL, low-density lipoprotein.

^*^Observed using a sex-stratified analysis to account for the OOA community’s male-only smoking cohort.

The minor allele (A) frequency of *PEAR1* rs12041331 in the HAPI Heart Study was 0.09, similar to that reported in other populations of European descent [7, 8, 10], resulting in 535 major allele homozygotes (GG), 101 heterozygotes (GA), and 5 minor allele homozygotes (AA), and conformed to expectations of Hardy-Weinberg equilibrium (P = 0.92). Characteristics of study participants by rs12041331genotype are shown in S1 Table. In our primary model accounting for clinical characteristics including age, sex, diabetes, SBP, DBP, and BMI, FMD was significantly higher in carriers of the *PEAR1* rs12041331 A-allele when compared to subjects who did not carry this allele (GG = 10.2 ± 0.3, GA = 10.8 ± 0.6, AA = 16.5 ± 5.4, P = 0.019). *PEAR1* rs12041331 remained significantly associated with FMD after also accounting for variation in D_base_ (GG = 10.2 ± 0.2, GA = 11.0 ± 0.4, AA = 14.1 ± 2.8, P = 0.032), HR (GG = 10.2 ± 0.1, GA = 11.1 ± 0.3, AA = 13.7 ± 2.3, P = 0.019), and both D_base_ and HR (GG = 10.2 ± 0.2, GA = 11.0 ± 0.4, AA = 13.9 ± 2.8, P = 0.034).

**Fig 1:**
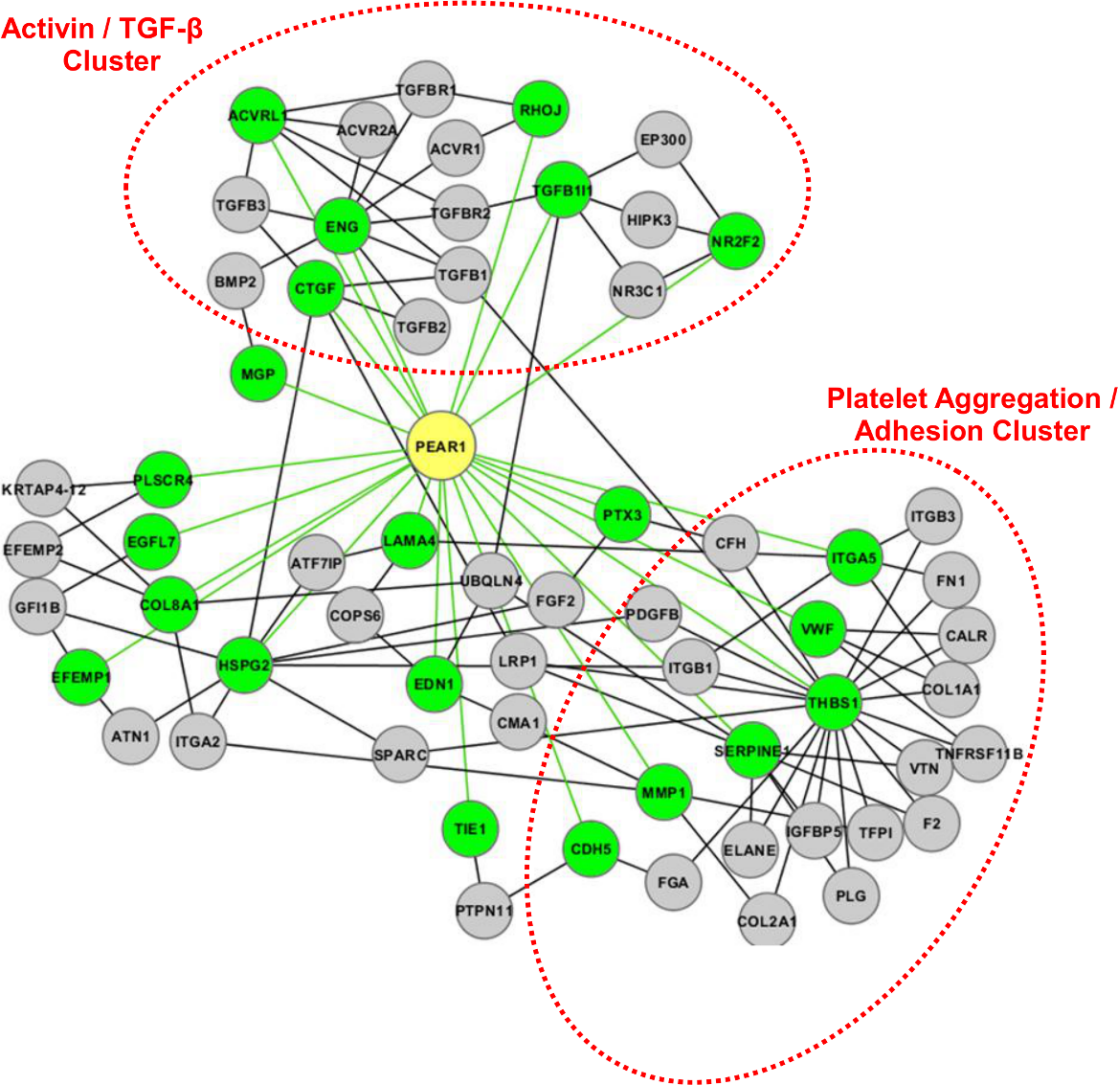
*PEAR1* Genetic Network. Genes highly correlated with *PEAR1* (green nodes) were evaluated for protein-protein interactions (gray nodes) that were shared by at least 2 of the 30 genes analyzed. Green lines indicate a co-expression relationship; black lines indicate a physical protein-protein interaction. Genetic neighborhoods of similar pathway or function have been highlighted and labeled.

Using the approach implemented in GAMMA, we identified genes that were most significantly associated with *PEAR1* expression (S2 Table) and built a genetic neighborhood of protein-protein interactions shared by the co-expressed genes (Fig 1). Of the 30 most significant co-expressed gene-pairs, using the 25 with MEDLINE entries, the top phenotypes predicted to be affected by changes in *PEAR1* gene expression were “endothelial cell migration,” “vasculogenesis,” and “angiogenesis” (Table 3). Similarly, the disease most highly predicted to be influenced by alterations in *PEAR1* gene expression was “vascular disease” (Table 3). Full lists of the phenotypes and diseases that were predicted to be influenced by *PEAR1* expression are shown in S3 and S4 Tables, respectively.

**Table 3:** Meta-Analysis Results of Publicly Available Microarray Datasets.

Top predicted phenotypes	# Shared Relations^*^	Score^†^
Endothelial cell migration	12	136
Vasculogenesis	11	103
Angiogenesis	20	86
Lymphangiogenesis	8	70
Neovascularization	11	57
Endothelial cell proliferation	9	55
Platelet aggregation	7	55
Cell adhesion	15	52
**Top predicted diseases**		
Vascular disease	12	59
Non-small cell lung carcinoma	14	55
Osteoarthritis	11	46
Preeclampsia	10	42
Pancreatic cancer	12	40
Diabetic nephropathy	9	39
Colorectal cancer	14	39

* Using the 25 genes with MEDLINE entries that are most highly correlated with *PEAR1* expression (see S2 Table), predicted phenotypes, diseases, and genes were identified with Global Microarray Meta-Analysis (GAMMA), and the number of shared relations represents how many of the 25 genes were related to term (left column).

^†^Score reflects the relative enrichment of observed connections within the analyzed network relative to a random network with the same number of connections per gene, enabling prediction to be prioritized [15].

**Fig 2:**
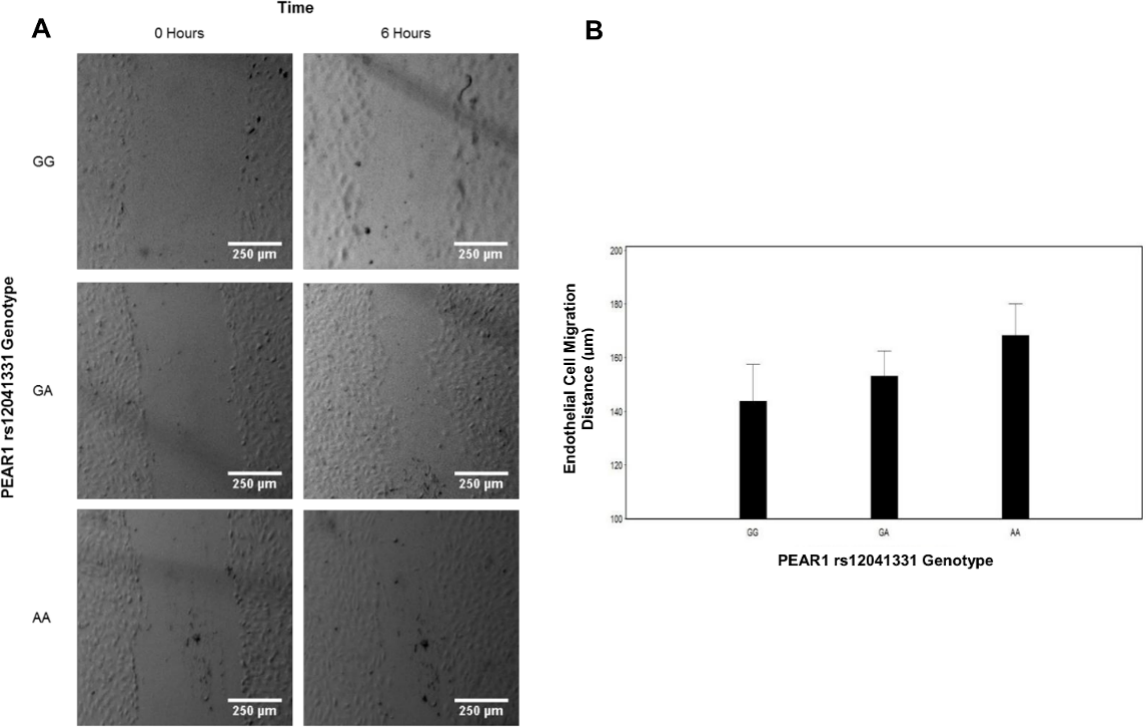
The Impact of *PEAR1* rs12041331 on Endothelial Cell Migration in Human Umbilical Vein Endothelial Cells (HUVECs). (A) Representative phase-contrast images of rs12041331-stratified HUVECs at 0 and 6 hours post-scratch generation during an *ex vivo* endothelial cell migration assay. Scale bar, 250 μm. (B) Quantitative depiction of mean HUVEC migration. Endothelial cell migration distance was calculated by dividing the area of the scratch by the height of the frame using ImageJ. Mean endothelial cell migration distance was calculated based on 72, 72, and 36 independent measurements for GG, GA, and AA genotypes, respectively, as described in the Materials and Methods section.

Given our GAMMA results, we functionally tested whether the well-described *PEAR1* rs12041331 variant significantly influenced endothelial cell migration. Genotypic differences in endothelial cell migration distances were assessed at 6 hours post-scratch generation. Consistent with the results of our microarray meta-analysis regarding endothelial cell migration, we observed that the A-allele of *PEAR1* rs12041331 was significantly associated with increased endothelial cell migration (P = 0.04; 143.8 ± 58.4 μm for the 4 GG cell lines, 153.1 ± 39.8 μm for the 4 GA cell lines, and 168.4 ± 34.8 μm for the 2 AA cell lines [Fig 2]). Moreover, this association remained statistically significant when we grouped cells line containing the A-allele (i.e. GA and AA genotypes) and compared them to GG homozygotes (P = 0.048; 143.8 ± 58.4 μm for the 4 GG cell lines and 158.2 ± 38.7 μm for cell lines that carried the A-allele [N = 6])

## Discussion

PEAR1 was identified in 2005 by Nanda and colleagues as a type I membrane protein that is highly expressed in platelets and endothelial cells, and is involved in platelet aggregation through secondary signaling via the αIIbβ3 integrin following platelet-platelet contact [1]. Several subsequent studies, including our own [10], have focused almost exclusively on the functional or phenotypic effects of this gene in platelets and megakaryocytes [1–3, 5–9, 11]. Taken together, these investigations have yielded insights into the molecular signaling of PEAR1 as a tyrosine kinase that signals through PI3K and Akt [1, 2], as well as the role of this receptor in megakaryopoiesis [3], platelet aggregation [1, 2, 5–11], antiplatelet therapy response [8–11], and cardiovascular events [10].

In this investigation, we measured FMD of the brachial artery, the most widely used noninvasive test of endothelial function, in order to estimate its heritability, establish clinical predictors, and assess the potential effect of genetic variation in *PEAR1* on endothelial function *in vivo*. In relatively healthy participants of the HAPI Heart Study, we found FMD to be normally distributed (S1 Fig). Gender and D_base_ accounted for over 45% of the variation in FMD, with age, smoking, SBP, DBP, hypertension, and HR jointly accounting for another 13–14%. Similar to previous reports [30, 31], our heritability estimates suggest that approximately 16% of the variation in FMD is attributable to relatedness, for which genetic factors likely contribute. To our knowledge, this investigation describes the first reported relationship between PEAR1 and clinical measures of endothelial function. Specifically, our data show that the minor allele of a common intronic variant (rs12041331) in this gene is significantly associated with greater brachial artery FMD. While the biological mechanism behind this association is not understood currently, future investigations probing the previously established relationship between PEAR1 and AKT signaling [2], an important mediator of FMD and nitric oxide production, seem warranted.

In an attempt to gain a more comprehensive view regarding phenotypes and diseases that may be influenced by PEAR1, we also used *in silico* genetic and statistical methodologies [12] to conduct a meta-analysis of publicly available microarray datasets. As expected, our *PEAR1* genetic network identified several genes critical in platelet adhesion and aggregation including von Willebrand factor (*vWF*), thrombospondin 1 (*THBS1*), and plasminogen activator inhibitor 1 (*SERPINE1*). Interestingly, we also identified several *PEAR1* co-expressed genes that are part of the TGF-β signaling system. These genes are important in blood vessel formation (*ACVRL1*) [32], endothelial cell migration and angiogenesis (*RhoJ*) [33], endothelial cell proliferation, smooth muscle cell recruitment, and vascular remodeling (*ENG*) [34, 35], as well as endothelial cell adhesion and survival (*CTGF*) [36]. Furthermore, several genes that were significantly co-expressed with *PEAR1* have also been previously implicated in endothelial-related pathological conditions including pulmonary fibrosis (*CTGF*) [37] and hemorrhagic telangiectasia (*ACVRL1* and *ENG*) [38]. While, to date, no investigation has evaluated the potential relationship between *PEAR1* and any of these genes and conditions, our results suggest that future studies may be warranted.

Through GAMMA, we also generated a list of phenotypes that are most likely to be influenced by changes in *PEAR1* expression. Interestingly, 6 of the 8 most significantly associated phenotypes predicted by GAMMA are critical in vascular endothelial function (endothelial cell migration, vasculogenesis, angiogenesis, neovascularization, endothelial cell proliferation, and cell adhesion). The two most significantly associated phenotypes that were not related to endothelial function were “platelet aggregation,” which has been previously validated [5–11], and “lymphangiogenesis,” which, at this time, has never been evaluated in the context of PEAR1. Similarly, a list of diseases predicted to be influenced by changes in *PEAR1* expression was generated using the same approach. Consistent with our previous report [10], “vascular disease” was most strongly related to changes in *PEAR1* expression. Preeclampsia, a condition characterized by high blood pressure and endothelial dysfunction, was also predicted to be influenced by PEAR1. Intriguingly, other diseases predicted to be related to PEAR1 included several types of cancer (e.g. colorectal, pancreatic, and non-small cell lung carcinoma), osteoarthritis, and diabetic nephropathy. While there is currently no available data suggesting a relationship between PEAR1 and osteoarthritis, diabetic nephropathy, aortic aneurisms, or cancer risk, it is interesting to speculate given the role of the TGF-β signaling system in the progression of these disorders.

In order to experimentally validate, in part, the results of our microarray meta-analysis, we tested whether a well-described genetic variant in *PEAR1* (rs12041331) significantly influenced endothelial cell migration, the most highly predicted phenotype to be affected by PEAR1 based on our GAMMA results, through the use of the well-described endothelial cell migration assay. Indeed, we observed that *PEAR1* rs12041331 was significantly associated with endothelial migration distance. Our results indicate that HUVECs homozygous for the A-allele of *PEAR1* rs12041331 have approximately 117% better endothelial cell migration capabilities compared to cells homozygous for the major allele (G). This novel observation not only strengthens our confidence in the results obtained by GAMMA, but also provides at least one phenotype by which PEAR1 influences endothelial cell biology.

To our knowledge, the only other investigation to date that has evaluated PEAR1 in the context of endothelial cell biology was a recently reported publication by Vandenbriele and colleagues [39]. In that study, it was observed that lentiviral-mediated knockdown of *PEAR1* resulted in enhanced endothelial cell proliferation through the Akt/p21/CDC2 pathway, and, consistent with the current investigation, observed that *PEAR1* knockdown resulted in increased endothelial cell migration, potentially suggesting that PEAR1 is a negative regulator of these phenotypes. Given that prior evidence has shown that PEAR1 influences AKT/PTEN signaling [2, 39], the increased endothelial migration observed with the rs12041331 minor allele as well as in *PEAR1* knockdown studies could suggest decreased action of PTEN, leading to an increase in AKT phosphorylation, a key mediator in endothelial cell migration. Given the observed impact of PEAR1 on endothelial cell proliferation and migration, the authors extended these findings showing that *PEAR1* knockdown also resulted in increased endothelial tube length, number, and branch points using a matrigel assay. Interestingly, they also showed that *Pear1* ^*-/-*^ mice have increased neoangiogenesis compared to wild-type mice using a hind limb ischemia ligation model as well as significantly decreased wound size and closure time in an independent skin wound healing model. Importantly, all of these findings are consistent with the aforementioned top phenotypes predicted to be associated with PEAR1 by GAMMA. Therefore, the data shown by Vandenbriele and colleagues, combined with our data showing that genetic variation in *PEAR1* influences flow-mediated dilation in humans suggest that PEAR1 may be a critical determinant of endothelial homeostasis with potential implications in the development of vascular disease.

Most of the investigations of PEAR1 to date have focused almost exclusively on its role in platelets and megakaryocytes. Indeed, prior mechanistic investigations by Kauskot and colleagues suggest that PEAR1 significantly influences sustained platelet aggregation through glycoprotein αIIbβ3 [2] as well as megakaryopoiesis through the PI3K/PTEN pathways [3]. In addition, several genetic investigations have shown that polymorphisms in *PEAR1* are associated with *ex vivo* platelet aggregation in response to several platelet agonists (e.g. ADP, collagen, epinephrine) as well as pre- and post-antiplatelet therapy treatment (i.e. aspirin and prasugrel) [5–11]. In our own previous investigation, we identified *PEAR1* rs12041331 as a strong determinant of collagen-stimulated platelet aggregation after dual anti-platelet therapy (DAPT) with aspirin and clopidogrel, as well as decreased 1-year survival in DAPT-treated patients undergoing percutaneous coronary intervention and increased rates of myocardial infarction (MI) in aspirin-treated patients with stable coronary artery disease [10]. However, despite the consistent data that shows PEAR1 significantly influences platelet biology and thrombosis, the current work and the investigation of Vandenbriele and colleagues highlight a novel genotype-phenotype relationship in the endothelium. Therefore, it is important that future investigations aiming to understand the relationship between this gene and cardiovascular disease take into account the pleiotropic nature of PEAR1 in these tissues.

There are some limitations to our study. While the use of FMD is the most commonly used method to non-invasively assess endothelial function, recent work by Atkinson and Batterham have shown that inadequate adjustment of FMD analyses, particularly of a ratio-scaling inconsistency between percent FMD and baseline vessel diameter, leads to biased results [40]. In order to minimize the effect of this potential confounder and others, we tested for association between *PEAR1* rs12041331 and FMD using several statistical models that adjusted for different clinical variables that impact FMD measurements (e.g. age, sex, D_base,_ and HR). In all models, *PEAR1* rs12041331 remained significantly associated with FMD. In addition, given the number of minor allele homozygotes (N = 5) and observed genotypic means, we also tested for association between rs12041331 and FMD using both dominant (GG vs AG/AA; P = 0.03) and recessive (GG/AG vs AA; P = 0.05) genetic models, showing a consistent direction of association. A potential limitation of the approach implemented in GAMMA is that it tends to be biased towards inclusion of genes that change significantly in their expression level and against genes whose transcriptional levels are too low to reliably detect. Although the analysis we performed of multiple genes for their published commonalities tends to reduce the bias towards a small number of genes skewing the results, the scientific literature itself may have its own biases in terms of preferences for what is both studied and reported. Therefore, while we functionally validated one phenotype predicted by GAMMA (i.e. endothelial migration), we believe future functional experiments to evaluate the relationship between *PEAR1* and the genes/phenotypes identified by GAMMA are warranted.

The dynamic relationship between platelets and endothelial cells is critical in cardiovascular physiology, and dysfunction of either cell type can lead to a cardiovascular event. There is a growing body of literature indicating that PEAR1 has important effects on platelet-related processes and cardiovascular outcomes. In this investigation, we have established for the first time that genetic variation in *PEAR1* significantly impacts endothelial function as well. Looking forward, it is critical to further characterize the function(s) of PEAR1 in both platelet and endothelial cells to elucidate the mechanism by which this gene may contribute to cardiovascular risk. Once understood, PEAR1 may be a novel target for treatment and prevention of cardiovascular disease.

## Supporting Information

S1 Fig Distribution of Flow-Mediated Dilation in 641 Amish Participants of the Heredity and Phenotype Intervention (HAPI) Heart Study. (PDF)

S1 Table Characteristics of HAPI Study Participants by rs12041331 Genotype. (PDF)

S2 Table Genes with expression most highly correlated with *PEAR1*. (PDF)

S3 Table Most highly predicted phenotypes for *PEAR1*. (PDF)

S4 Table Predicted diseases relevant to *PEAR1* Dysregulation. (PDF)

S5 Table Raw endothelial cell migration data. (XLSX)
