# Supplementary material for: Genetic Variation in the Platelet Endothelial Aggregation Receptor 1 Gene Results in Endothelial Dysfunction
Source: PLoS One. 2015 Sep 25;10(9):e0138795. doi: 10.1371/journal.pone.0138795 (PMC4583223; doi:10.1371/journal.pone.0138795)
Supplement: S1 Fig — (PDF) [file pone.0138795.s001.pdf]

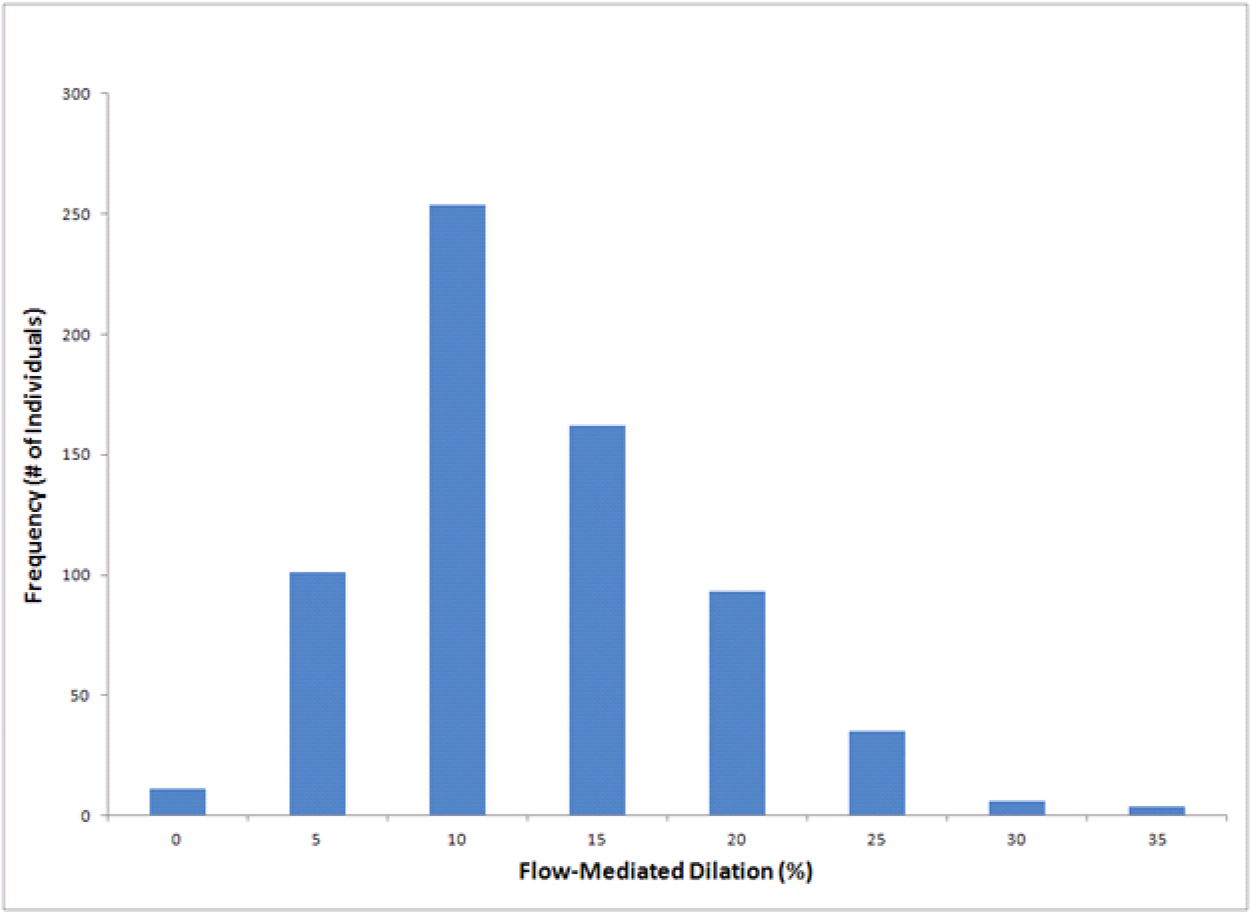

S1 Fig. Distribution of Flow-Mediated Dilation in 641 Amish Participants of the Heredity and Phenotype Intervention (HAPI) Heart Study.
