# Supplementary material for: Genetic Variation in the Platelet Endothelial Aggregation Receptor 1 Gene Results in Endothelial Dysfunction
Source: PLoS One. 2015 Sep 25;10(9):e0138795. doi: 10.1371/journal.pone.0138795 (PMC4583223; doi:10.1371/journal.pone.0138795)
Supplement: S1 Table — (PDF) [file pone.0138795.s002.pdf]

S1 Table. Characteristics of HAPI Study Participants by rs12041331 Genotype.

	PEAR1 rs12041331 Genotype		
**Characteristic (Units)**	**GG**	**GA**	**AA**
Number (n)	535	101	5
Age ± SD (years)	43.2 ± 13.9	43.6 ± 13.8	33.0 ± 17.2
BMI ± SD (kg/m^2)	26.2 ± 4.0	26.9 ± 4.3	26.9 ± 2.9
No. of female gender (%)	232 (43.4)	41 (40.6)	3 (60.0)
Brachial artery width pre-occlusion ± SD (mm)	3.8 ± 3.1	3.7 ± 0.6	3.4 ± 0.6
Brachial artery width post-occlusion ± SD (mm)	4.2 ± 3.3	4.1 ± 0.6	3.9 ± 0.6
Systolic blood pressure ± SD (mm Hg)	121.5 ± 14.4	120.3 ± 14.7	123.0 ± 11.7
Diastolic blood pressure ± SD (mm Hg)*	77.2 ± 8.6	75.6 ± 8.7	68.4 ± 4.2
No. with hypertension (%)	71 (13.3)	11 (10.9)	0 (0)
Total cholesterol ± SD (mg/dl)	207.2 ± 45.7	205.4 ± 51.7	183.6 ± 18.4
LDL cholesterol ± SD (mg/dl)	138.1 ± 42.5	136.6 ± 47.2	124.4 ± 17.8
HDL cholesterol ± SD (mg/dl)	55.9 ± 13.9	55.3 ± 14.4	50.0 ± 5.6
Triglycerides ± SD (mg/dl)	66.2 ± 41.1	67.7 ± 44.5	45.8 ± 15.4
No. with hypercholesteremia (%)	94 (17.7)	14 (13.9)	0 (0)
No. with self-reported diabetes (%)	3 (0.6)	2 (2.0)	0 (0)
Hematocrit ± SD (%)	41.1 ± 3.4	41.3 ± 3.1	39.6 ± 5.8
White blood cell count ± SD (n x 1000)	5.3 ± 1.2	5.2 ± 1.2	4.8 ± 1.3
Platelet count ± SD (n x 100,000)	235.0 ± 51.8	236.5 ± 50.9	259.4 ± 57.4
No. of current smokers (%)†	56 (10.6)	17 (17.2)	0 (0)
No. taking aspirin (%)	14 (2.6)	5 (5.0)	0 (0)
No. taking lipid-lowering medications (%)	5 (0.9)	2 (2.0)	0 (0)
No. taking anti-hypertensive medications (%)	1 (0.2)	0 (0)	0 (0)

* Diastolic blood pressure was significantly different between genotypes (p=0.02) .No other traits were significantly different by genotype.

† Observed using a sex-stratified analysis to account for the OOA community's male-only smoking cohort.
