# Supplementary material for: Genetic Variation in the Platelet Endothelial Aggregation Receptor 1 Gene Results in Endothelial Dysfunction
Source: PLoS One. 2015 Sep 25;10(9):e0138795. doi: 10.1371/journal.pone.0138795 (PMC4583223; doi:10.1371/journal.pone.0138795)
Supplement: S2 Table — (PDF) [file pone.0138795.s003.pdf]

S2 Table. Genes with expression most highly correlated with *PEAR1.*

Gene	Full Name	Primary Localization
CDH5	cadherin 5, type 2 (vascular endothelium)	Plasma membrane
ADCY4	adenylate cyclase 4	Plasma membrane
FAM43A	family with sequence similarity 43, member A	Unknown
MFRP	membrane frizzled-related protein	Plasma membrane
C1QTNF5	C1q and tumor necrosis factor related protein 5	Extracellular
TIE1	tyrosine kinase with immunoglobulin-like and EGF-like domains 1	Plasma membrane
COL8A1	collagen, type VIII, alpha 1	Extracellular
HHIP-AS1	HHIP antisense RNA 1	Unknown
LDLRAD2	low density lipoprotein receptor class A domain containing 2	Plasma membrane
FOXC2	forkhead box C2 (MFH-1, mesenchyme forkhead 1)	Nucleus
MGP	matrix Gla protein	Extracellular
WTAPP1	Wilms tumor 1 associated protein pseudogene 1	Unknown
RHOJ	ras homolog family member J	Endosome
ROBO4	roundabout, axon guidance receptor, homolog 4 (Drosophila)	Plasma membrane
ACVRL1	activin A receptor type II-like 1	Plasma membrane
SERPINE1	serpin peptidase inhibitor, clade E, member 1	Extracellular
SULF1	sulfatase 1	Extracellular
MMRN1	multimerin 1	Extracellular
HSPG2	heparan sulfate proteoglycan 2	Extracellular
ECSCR	endothelial cell surface expressed chemotaxis and apoptosis regulator	Plasma membrane
BTBD19	BTB (POZ) domain containing 19	Unknown
HYI	hydroxypyruvate isomerase (putative)	Nucleus
MMP1	matrix metallopeptidase 1 (interstitial collagenase)	Extracellular
VWF	von Willebrand factor	Extracellular
LAMA4	laminin, alpha 4	Extracellular
HHIP	hedgehog interacting protein	Plasma membrane
EDN1	endothelin 1	Extracellular
PTX3	pentraxin 3, long	Extracellular
LOC392536	filamin binding LIM protein 1 pseudogene	Unknown
TGFB1I1	transforming growth factor beta 1 induced transcript 1	Nucleus
BCL6B	B-cell CLL/lymphoma 6, member B	Nucleus
SPHK1	sphingosine kinase 1	Cytoplasm
ENG	endoglin	Plasma membrane

TNFRSF10D	tumor necrosis factor receptor superfamily, member 10d, decoy with truncated death domain	Plasma membrane
VWFP1	von Willebrand factor pseudogene 1	Unknown
MMRN2	multimerin 2	Extracellular
ESM1	endothelial cell-specific molecule 1	Extracellular
EFEMP1	EGF containing fibulin-like extracellular matrix protein 1	Extracellular
GLCE	glucuronic acid epimerase	Golgi apparatus
LOC101928281	uncharacterized LOC101928281	Unknown
CTGF	connective tissue growth factor	Extracellular
NR2F2	nuclear receptor subfamily 2, group F, member 2	Nucleus
ITGA5	integrin, alpha 5 (fibronectin receptor, alpha polypeptide)	Plasma membrane
PLSCR4	phospholipid scramblase 4	Plasma membrane
GPR126	G protein-coupled receptor 126	Plasma membrane
THBS1	thrombospondin 1	Extracellular
ANKRD1	ankyrin repeat domain 1 (cardiac muscle)	Nucleus
ROBO3	roundabout, axon guidance receptor, homolog 3 (Drosophila)	Plasma membrane
EGFL7	EGF-like-domain, multiple 7	Extracellular
