# Supplementary material for: Genetic Variation in the Platelet Endothelial Aggregation Receptor 1 Gene Results in Endothelial Dysfunction
Source: PLoS One. 2015 Sep 25;10(9):e0138795. doi: 10.1371/journal.pone.0138795 (PMC4583223; doi:10.1371/journal.pone.0138795)
Supplement: S3 Table — (PDF) [file pone.0138795.s004.pdf]

S3 Table. Most highly predicted phenotypes for *PEAR1*.

Phenotype	# of Shared Relationships	Score
Endothelial cell migration	12	136
Vasculogenesis	11	103
Angiogenesis	20	86
Lymphangiogenesis	8	70
Neovascularization	11	57
Endothelial cell proliferation	9	55
Platelet aggregation	7	55
Cell adhesion	15	52
Paracrine Signaling	13	51
Cell migration	14	51
Embryonic development	14	47
Focal adhesions	9	45
Dermal fibroblasts	9	42
Hypoxia-inducible	10	41
Endothelial cell surface	6	40
Cell motility	10	38
Endothelial cell growth	6	38
Transdifferentiation	7	37
DNA methylation	11	37
Tyrosine phosphorylation	11	36
Leukocyte extravasation	5	36
Signal transduction	15	36
Retinal endothelial cell	5	35
Carotid artery	11	34
Chemotaxis	10	34
Wound healing	11	34
Angiogenesis inhibitors	7	33
Angiogenic phenotype	5	33
Cell proliferation	16	33
Nuclear translocation	10	32
Signal transduction pathways	11	32
Cell differentiation	12	31
Regulation of angiogenesis	5	30
Heparin binding	7	30
Clopidogrel	5	29
Response to LPS	7	29
Tissue homeostasis	7	29
Adiponectin levels	6	29
Morphogenesis	10	29
Sprouting angiogenesis	4	28

Capillary morphogenesis	4	28
Glomerular filtration rate	7	19
Phagocytosis	8	18
