# Supplementary material for: Genetic Variation in the Platelet Endothelial Aggregation Receptor 1 Gene Results in Endothelial Dysfunction
Source: PLoS One. 2015 Sep 25;10(9):e0138795. doi: 10.1371/journal.pone.0138795 (PMC4583223; doi:10.1371/journal.pone.0138795)
Supplement: S4 Table — (PDF) [file pone.0138795.s005.pdf]

**S4 Table. Predicted diseases relevant to *PEAR1* Dysregulation.**

| Disease | # of Shared Relationships | Score |
|---|---|---|
| Vascular disease | 12 | 59 |
| Non-small cell lung carcinoma | 14 | 55 |
| Osteoarthritis | 11 | 46 |
| Preeclampsia | 10 | 42 |
| Pancreatic cancer | 12 | 40 |
| Diabetic nephropathy | 9 | 39 |
| Colorectal cancer | 14 | 39 |
| Lymph node metastasis | 9 | 37 |
| Melanoma | 13 | 36 |
| Breast cancer | 16 | 35 |
| Coronary artery disease | 12 | 33 |
| MDA-MB-231 breast cancer | 6 | 33 |
| Metastatic disease | 11 | 33 |
| Intimal hyperplasia | 6 | 33 |
| Mammary tumor | 11 | 33 |
| Chronic kidney disease | 9 | 32 |
| Gastric cancer | 11 | 32 |
| Pulmonary arterial hypertension | 7 | 32 |
| Atherosclerotic plaque | 7 | 32 |
| Systemic sclerosis | 8 | 31 |
| Angiosarcoma | 6 | 31 |
| Insulin resistance | 11 | 30 |
| Renal disease | 10 | 30 |
| Liver fibrosis | 8 | 29 |
| Proliferative diabetic retinopathy | 5 | 29 |
| Myocardial infarction | 11 | 28 |
| Kidney disease | 8 | 27 |
